# Visual rating scales of atrophy differentiate sporadic behavioral variant frontotemporal dementia from primary psychiatric disorder: DIPPA study

**DOI:** 10.1007/s00702-026-03223-y

**Published:** 2026-07-02

**Authors:** Giorgio Giulio Fumagalli, Chiara Fornari, Sterre C. M. de Boer, Chiara Fenoglio, Andrea Arighi, Lina Riedl, Ramon Landin-Romero, Sophie Matis, Ishana Rue, Zac Chatterton, Janine Diehl-Schmid, Glenda Halliday, Simon Ducharme, Olivier Piguet, Daniela Galimberti, Yolande Pijnenburg

**Affiliations:** 1 Center for Mind/Brain Sciences (CIMeC), University of Trento, Corso Bettini 31, 38068 Rovereto, Italy; 2 Alzheimer Center Amsterdam, Neurology, Vrije Universiteit Amsterdam, Amsterdam UMC location VUmc, Amsterdam, The Netherlands; 3 Amsterdam Neuroscience, Neurodegeneration, Amsterdam, The Netherlands; 4 Ospedale Maggiore Policlinico, Neurodegenerative Diseases Unit, Fondazione IRCSS Ca’ Granda, Milan, Italy; 5 Department of Biomedical, Surgical and Dental Sciences, Centro Dino Ferrari, University of Milan, Milan, Italy; 6 School of Medicine, Department of Psychiatry and Psychotherapy, Technical University of Munich, Munich, Germany; 7 Sydney School of Health Sciences, Faculty of Medicine and Health, The University of Sydney, Sydney, Australia; 8 Brain and Mind Centre, The University of Sydney, Sydney, Australia; 9 Department of Psychiatry, Douglas Mental Health University Institute, McGill University, Montreal, QC Canada; 10 Clinical Center for Psychiatry, Psychotherapy, Psychosomatic Medicine, Geriatrics and Neurology, kbo-Inn-Salzach-Klinikum, Wasserburg/Inn, Germany; 11 McConnell Brain Imaging Centre, Montreal Neurological Institute, McGill University, Montreal, QC Canada; 12 School of Psychology, The University of Sydney, Sydney, Australia

**Keywords:** Frontotemporal dementia, Dementia, Visual rating scales, Atrophy, Biomarkers, Magnetic resonance imaging, Psychiatric disorders

## Abstract

Sporadic behavioral variant frontotemporal dementia (bvFTD) is often misdiagnosed as late-onset primary psychiatric disorder (PPD) due to overlapping symptoms and lack of disease-specific biomarkers. This multicenter pilot study aimed to identify brain atrophy patterns using visual rating scales (VRS) that distinguish between groups, and to compare VRS performance with standard clinical assessment. Magnetic resonance images from bvFTD and PPD patients across five centers were retrospectively reviewed. One rater, blinded for the clinical diagnosis, applied eight VRS. Group differences were assessed, the most predictive scales were identified and combined into a composite score, and the predictivity compared. 297 bvFTD and 92 PPD patients were analysed. All VRS yielded higher atrophy in bvFTD than in PPD patients. The Orbitofrontal, the Anterior-Temporal, and the Fronto-Insula scales were the strongest discriminators, and their composite score outperformed any individual scale. VRS may provide useful diagnostic support for distinguishing bvFTD from PPD.

**Supplementary Information:**

The online version contains supplementary material available at https://doi.org/10.1007/s00702-026-03223-y.

## Background

Late-onset behavioral and personality changes in adulthood are a complex clinical phenomenon (Krudop et al. 2015) with multiple possible etiologies, encompassing both primary psychiatric disorders (PPD), including schizophrenia, major depressive disorder, bipolar disorder, obsessive-compulsive disorder, autism spectrum disorders and some personality disorders, and neurodegenerative disorders, such as the frontotemporal dementia (FTD) (Ducharme et al. 2015). FTD represents a heterogeneous spectrum of clinical syndromes, including the behavioral variant (bvFTD), the language variants of primary progressive aphasia, the right temporal variant, and it is also frequently associated with motor neuron diseases [for a review: (Antonioni et al. 2023)]. Notably, establishing an accurate differential diagnosis between bvFTD and PPD remains challenging due the overlapping clinical symptoms and the lack of validated biomarkers (Ducharme et al. 2020). Nevertheless, distinguishing between the two groups of disorders is of crucial importance, as they require different pharmacological treatment and rehabilitation programs, and early intervention significantly influences the prognosis of both conditions.

The identification of genetic mutations causative of FTD in 10–30% of patients (Olszewska et al. 2016, Sirkis et al. 2019), may offer valuable diagnostic and prognostic information. However, the absence of a reliable non-genetic FTD biomarker limits the applicability of this approach to sporadic cases. Another potential discriminative marker between the two conditions has been identified in the neurofilament light chain (de Boer et al. 2025). However, its assessment is not widely available yet and may be limited by comorbidities and the lack of a harmonized reference standard. Structural magnetic resonance imaging (MRI) remains a critical component of the diagnostic investigation in patients with adult-onset behavioral changes and should be prioritized before more advanced imaging techniques (Soucy et al. 2013). Although the presence of frontal and anterior temporal atrophy increases the diagnostic specificity for bvFTD (Rascovsky et al. 2011), allowing clinicians to upgrade the diagnostic certainty from *possible* to *probable* according to the current bvFTD criteria, neuroradiological evaluation alone is often insufficient for a definitive diagnosis. This is especially frequent in the early stages of the disease, when atrophy may not be extensive and so not yet distinguishable from the age-related volumetric changes with standard visual radiological inspection of images (Vijverberg et al. 2016, Chow et al. 2008, Gregory et al. 1999). Indeed, non-specific brain volume loss is common in major psychiatric disorders, such as ventricular enlargement in schizophrenia and hippocampal atrophy in major depression, but these findings are primarily based on group-level statistics, and the magnitude of change is not sufficient to be reliably detected individual differences (Selvaraj et al. 2012).

Therefore, identifying biomarkers to distinguish between bvFTD and PPD remains a clinically relevant challenge. The multicenter DIPPA-FTD study was designed to address this issue by developing diagnostic and prognostic tools capable of distinguishing sporadic bvFTD from late-onset PPD in its earliest stages (de Boer et al. 2024). Among the data collected in this protocol, structural MRI provides insights on the utility of neuroimaging in differentiating between bvFTD and psychiatric diseases (see also (Krudop et al. 2016)), and thus offers an opportunity to explore clinically applicable methods that leverage already available imaging. One pragmatic approach may be the systematic evaluation of brain atrophy through the visual rating scales. These scales have the advantage of being quick, cost-effective, and applicable in standard clinical settings. Notably, the visual rating scales have already demonstrated utility and widespread availability within the FTD dementia spectrum, for example in differentiating genetic variants mutations (Fumagalli et al. 2018), and in distinguishing among Primary Progressive Aphasia (PPA) syndromes (Falgàs et al. 2024, Fornari and Fumagalli 2025). However, their potential in the differential diagnosis between bvFTD and PPD remains underexplored.

The aim of this pilot study was to evaluate the utility of visual rating scales of atrophy in the differential diagnosis between late-onset PPD and sporadic bvFTD. Specifically, the study aimed to (a) assess the overall utility of visual rating scales, (b) identify which individual scales are the strongest discriminators between sporadic bvFTD and late-onset PPD, (c) compare the performance of visual rating scales with a standard radiological inspection, and (d) determine whether a combination of scales provides improved diagnostic accuracy.

## Methods

### Participants

Participants were retrospectively recruited from 5 different centres: Alzheimer center Amsterdam; Brain and Mind Centre of the University of Sydney; the Douglas Mental Health University Institute (McGill University); Fondazione Ca’ Granda, IRCCS Ospedale Maggiore Policlinico (University of Milan); and the Technical University of Munich. Among patients recruited in the DIPPA-FTD retrospective study (de Boer et al. 2024), only those with neurological examination, clinical history, neuropsychological evaluation, and T1-weighted 3D MRI available were included. When clinically indicated, functional neuroimaging (with FDG-PET or SPECT) and amyloid biomarkers (with cerebrospinal fluid or Amyloid-PET) were performed. All participants provided informed written consent to participate in the clinical research.

As previously described (de Boer et al. 2025), the retrospective DIPPA-FTD study (de Boer et al. 2024) enrolled participants aged 45 years and older referred with late-onset behavioral change, eventually diagnosed with either sporadic bvFTD or late-onset PPD. PPD patients were classified according to one of the DSM-5 criteria for major depressive disorder, schizophrenia, delusional disorder, bipolar disorder, manic episode, personality disorders, or obsessive-compulsive disorder (American Psychiatric Association 2013). Participants diagnosed with PPD were enrolled only if clinicians confirmed the diagnosis, and if the screening for *C9orf72* repeat expansion was negative. Patients with bvFTD were included if they fulfilled current diagnostic criteria for probable or definite bvFTD (pathology-based) (Rascovsky et al. 2011) with a Clinical Dementia Rating (Morris 1993) score of ≤ 1. The diagnosis of sporadic bvFTD was defined by a Goldman score of ≥ 3 (Goldman et al. 2005, Goldman et al. 2011), or a Wood classification of ‘Low’ or ‘Apparent sporadic’ (Wood et al. 2013) in the absence of *C9orf72* repeat expansion. bvFTD patients with a Goldman score of 2 or a Wood classification of ‘Medium’ were included only if genetic testing for *C9orf72*,* GRN* and *MAPT* mutations were negative. Lastly, bvFTD patients were excluded if the Goldman score was 1 and/or the Wood score ‘High’. Patients were also excluded whether the Mini-Mental State Examination (MMSE) score was lower than 18 points, the Clinical Dementia Rating (CDR) scale higher than 2, whether the patients had history of alcohol abuse, and whether positive Alzheimer’s disease biomarkers were detected (de Boer et al. 2024).

### Magnetic resonance imaging (MRI)

#### MRI acquisition

Whole brain T1-weighted structural MRI were collected at multiple centers using standard acquisition protocols. Details of the MRI scanners and parameters at each center are reported in Table S1 (Supplementary Material). All images underwent visual quality control to check for artifacts and brain abnormalities.

#### Visual rating protocol

A protocol of visual rating scales of atrophy (Figure S1, Supplementary Material), as described in previously published papers (Fumagalli et al. 2018, Falgàs et al. 2024, Harper et al. 2016, Fumagalli et al. 2020), was applied independently by one rater (GF, a neurologist with previous experience of visual rating in dementia) blind for all the demographic and clinical information. In particular, the scales used in the protocol were: Orbitofrontal (OF), Anterior cingulate (AC), Anterior Temporal (AT), Fronto-insula (FI), Medial Temporal (MTA), and Posterior scale (PA).

Briefly, OF and AC scales, that evaluate respectively olfactory sulcus and cingulate sulcus, are rated in the coronal plane on the most anterior slice where the corpus callosum becomes visible with a four-part grading system: grade 0, representing no atrophy (no cerebrospinal fluid [CSF] visible within the sulcus); grade 1, mild widening of the sulcus (CSF just becomes visible); grade 2, moderate widening; and grade 3, severe widening (with the sulcus assuming a triangular shape). The AT scale looked at the aspects of the temporal pole in coronal view, using a 5-point system: grade 0 representing normal appearances, grade 1 only slight prominence of anterior temporal sulci, grade 2 definite widening of the temporal sulci, grade 3 severe atrophy and ribbon-like nature of the gyri, and grade 4 a simple linear profile of the temporal pole. The FI scale is a 4-point scale evaluating the circular sulcus of the insula in the coronal view on the slice where the anterior commissure becomes visible and the two following posterior. The MTA is a 5-point grade scale that looks at the medial temporal lobe in coronal view: grade 0 is normal; grade 1 a widened choroidal fissure; grade 2 an increased widening of the choroidal fissure, widening of temporal horn and opening of other sulci; grade 3 pronounced volume loss of the hippocampus; and grade 4 end-stage atrophy. PA scale is a 4-point scale evaluating posterior cortical atrophy using three views (coronal, axial and sagittal): grade 0 representing closed posterior cingulate and parieto-occipital sulci; grade 1 mild widening of the posterior cingulate and parieto-occipital sulci, with mild atrophy of the parietal lobes and precuneus; grade 2 substantial widening of the posterior cingulate and parieto-occipital sulcus, with substantial atrophy of the parietal lobes and precuneus; and grade 3 end-stage atrophy with evident widening of both sulci and knife-blade atrophy of the parietal lobes and precuneus.

Two 4-point visual rating scales for the ventricular enlargement in-house implemented were included in the protocol. The Mean Ventricular scale (MV) was assessed in coronal view on the first slice where the anterior commissure was visible, by evaluating the angle of the superior portion of lateral ventricles: grade 0 representing normal ventricles, acute angle; grade 1 mild enlargement with a slightly widened angle; grade 2 moderate enlargement with a more obtuse angle; grade 3 marked dilatation with a wide angle and rounded ventricular contours. The Axial Ventricular (AV) was assessed in axial view, by scrolling through the brain and evaluating the enlargement of the anterior lateral ventricles: grade 0 normal-sized ventricles; grade 1 mild widening of the frontal horns; grade 2 moderate dilatation with evident enlargement of frontal horns; grade 3 severe dilatation, with markedly enlarged ventricles.

To increase rating consistency, reference images for each scale were provided (Figure S1, Supplementary Material) and the images were presented in random order. For all the scales, right and left sides were rated separately and then a mean score of the two hemispheres was calculated.

Furthermore, based on the standard radiological inspection, the rater was asked to provide a diagnostic impression by deciding whether the patient should be classified in the PPD or FTD group. This judgment was made after evaluating the participant’s MRI scans using visual rating scales, and the resulting classification reflects the rater’s summary decision, combining the MRI inspection with the clinical expertise of disease patterns.

Lastly, the rater re-rated a subset of 37 randomly chosen images, representing 9.5% of the total images set included in the analysis, to assess the intra-rater reliability for both the visual rating scales and the diagnostic impression.

The software used to display images was MRIcroGL (https://www.nitrc.org/projects/mricrogl/) (Rorden and Brett 2000, Rorden et al. 2007). The images were rated in the native space, in keeping with standard clinical reads.

### Statistical analyses

The two groups were compared for demographic characteristics. Categorical data were reported as frequency and were analysed using the Chi-Squared Test (χ^2^), and quantitative data were presented as mean and standard deviation (sd). Differences of the quantitative data between the patients (2 levels: bvFTD, PPD) were tested using the Student’s T-test (T) or the non-parametric Mann-Whitney test (U) when the normality (Shapiro-Wilk test) was violated or the Welch test when the equality of variance assumption (Levene’s test) was violated. The differences between the groups in the Mini-Mental State Examination (MMSE) was assessed using the analysis of covariance (ANCOVA), controlling for age at the visit and education.

The intra-rater agreement for VRS and for the classification made by the rater’s diagnostic impression was assessed using the weighted Cohen’s Kappa. Values below 0.2 indicated poor agreement, a range from 0.21 to 0.4 indicated fair agreement, a range from 0.41 to 0.6 indicated a moderate agreement, a range from 0.61 to 0.8 indicated a substantial agreement, and a range from 0.81 to 1 a near perfect to perfect agreement (Fleiss et al. 2003). Rater accuracy for the standard diagnostic impression, namely the classification of patients into the bvFTD or PPD group, was calculated as the proportion of correctly classified cases relative to the total number of patients.

Group differences in the VRS scores were assessed using regression models. Exploratory ordinal logistic regression analyses of hemispheric-specific VRS scores (left and right) were performed to assess potential lateralized effects. The primary analyses were conducted using linear regression models on the mean bilateral scores. Diagnostic group (PPD vs. bvFTD) was included as the main predictor, with disease duration and sex as predictors. Given the number of VRS analysed, p-values were adjusted for multiple comparisons using the Holm–Bonferroni method.

To evaluate the contribution of age at onset and MMSE score to group classification independently of imaging-based measures, a logistic regression model was fitted including group membership as the outcome.

To verify whether group differences in the mean VRS scores were independent of clinical status, additional linear regression models were conducted including MMSE score and disease duration as covariates.

In linear regression models, statistical significance was evaluated using ANOVA (F-tests), and results were reported as regression coefficients (β) with corresponding standard errors, t-values, and p-values, and the model fit was assessed using R².

The diagnostic performance of each scale was evaluated using the Receiver Operating Characteristic curve (ROC) analysis, and the area under the curve (AUC) was computed. AUC values below 0.6 indicated a failed model, values between 0.6 and 0.7 indicated poor discrimination, values between 0.7 and 0.8 indicated fair discrimination, values between 0.8 and 0.9 indicated good discrimination, and values between 0.9 and 1 indicated excellent discrimination ability (de Hond et al. 2022).

To derive an optimal predictive model, a logistic regression with forward stepwise selection was performed, including VRS mean scores as predictors, diagnostic group as the outcome, sex as a factor, and age at MRI as a covariate. A composite score was computed by summing the scales retained in the final model. ROC analysis was then applied to the composite score, and optimal cut-off values were identified using the Youden index. Pairwise comparisons of AUC values were performed using DeLong’s test (DeLong et al. 1988) to identify the best-performing model.

All the analyses were conducted using Jamovi (version 2.4.8.) (The Jamovi 2023, R Core Team 2022, Thiele 2019, Friesen et al. 2019, Stites and Wilen 2020, Serdar 2022, Fox and Weisberg 2020, Sing et al. 2015), and R (v. 4.3.1) with the ordinal, lm4, lmerTest, pROC, and ggplot2 packages. A two-tail p-value < 0.05 was considered statistically significant.

## Results

### Demographic

A total of 389 patients were included in the study: 297 bvFTD and 92 PPD. The PPD group comprised a heterogeneous set of psychiatric diagnoses, predominantly major depressive disorder (*n* = 66), along with bipolar disorder (*n* = 8), delusional disorder (*n* = 4), schizoaffective disorder (*n* = 2), maniac episode (*n* = 1), schizophrenia (*n* = 1), and other psychiatric diagnosis (*n* = 10). The bvFTD group included 287 probable bvFTD, 1 Frontotemporal Dementia with Motor Neuron Disease (FTD-MND), and 9 autopsy-defined bvFTD. The two groups were comparable in terms of sex, education, and disease duration (i.e., the difference between the age at the MRI and the age at onset), but the PPD group had significantly lower age at onset, at MRI, at the first visit, and higher MMSE score (Table 1).

**Table 1:** Demographic characteristics of the sample

*N* (M/F)	bvFTD	PPD	Statistics
	297 (169/128)	92 (60/32)	χ^2^ = 2.01, *p* = 0.157
Mean Education in years (sd)	12.1 (3.53)	11.7 (3.48)	U = 7836, *p* = 0.443
Mean Age at onset in years (sd)	60.3 (8.81)	54.8 (8.52)	T_(371)_ = 5.22, *p* < 0.001
Mean Age at first visit in years (sd)	64.4 (8.39)	58.9 (7.67)	T_(280)_ = 5, *p* < 0.001
Mean Age at MRI in years (sd)	64.2 (8.40)	59.0 (8.07)	U = 8897, *p* < 0.001
Mean MMSE score (sd)	24.7 (4.3)	26.3 (3.7)	F_(1,154)_ = 9.17, *p* = 0.003
Mean Disease duration in years (sd)	4.30 (4.29)	3.81 (3.26)	T_(117.8)_ = 0.979, *p* = 0.329

Sample Numerosity (N); Male (M); Female (F); Mini-Mental State Examination (MMSE); standard deviation (sd); Chi-squared test (χ^2^); Mann-Whitney test (U); Student T-Test (T); Analysis of covariance (F).

### Diagnostic impression

**Fig. 1:**
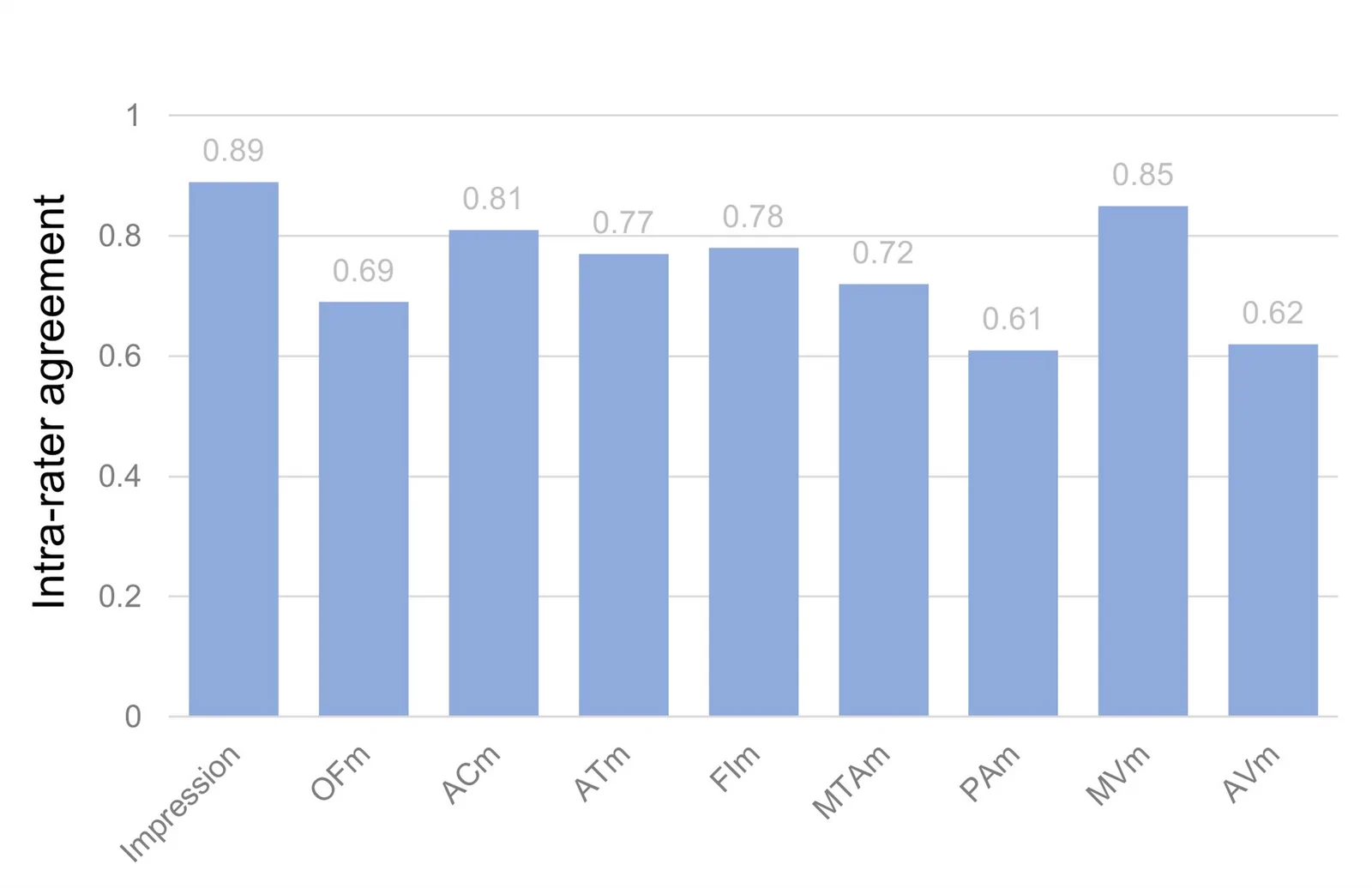
Intra-rater agreement (on the y-axis) both for the rater’s diagnostic impression and for each visual rating scale (OFm: mean Orbitorontal; ACm: mean Anterior Cingulate; ATm: mean Anterior Temporal; FIm: mean Fronto-Insula; MTAm: mean Medial Temporal; PAm: mean Posterior; MVm: mean Mean Ventricular; AVm: mean Axial Ventricular)

The intra-rater agreement for the diagnostic impression (i.e., rater’s judgment whether a participant belonged to the bvFTD or PPD group) showed a weighted Kappa score of 0.89 (Fig. 1). Furthermore, the rater accurately predicted the correct diagnosis with the standard radiological inspection in 76.9% of the cases with a sensitivity of 75.4% and a specificity of 81.5%.

### Visual rating scales

#### Intra-rater agreement

All scales demonstrated substantial (OFm, ATm, FIm, MTAm, PAm, AVm) to excellent (MVm, ACm) intra-rater reliability with a weighted Kappa score, indicating high consistency across repeated ratings (Fig. 1).

#### Groups comparison

**Fig. 2:**
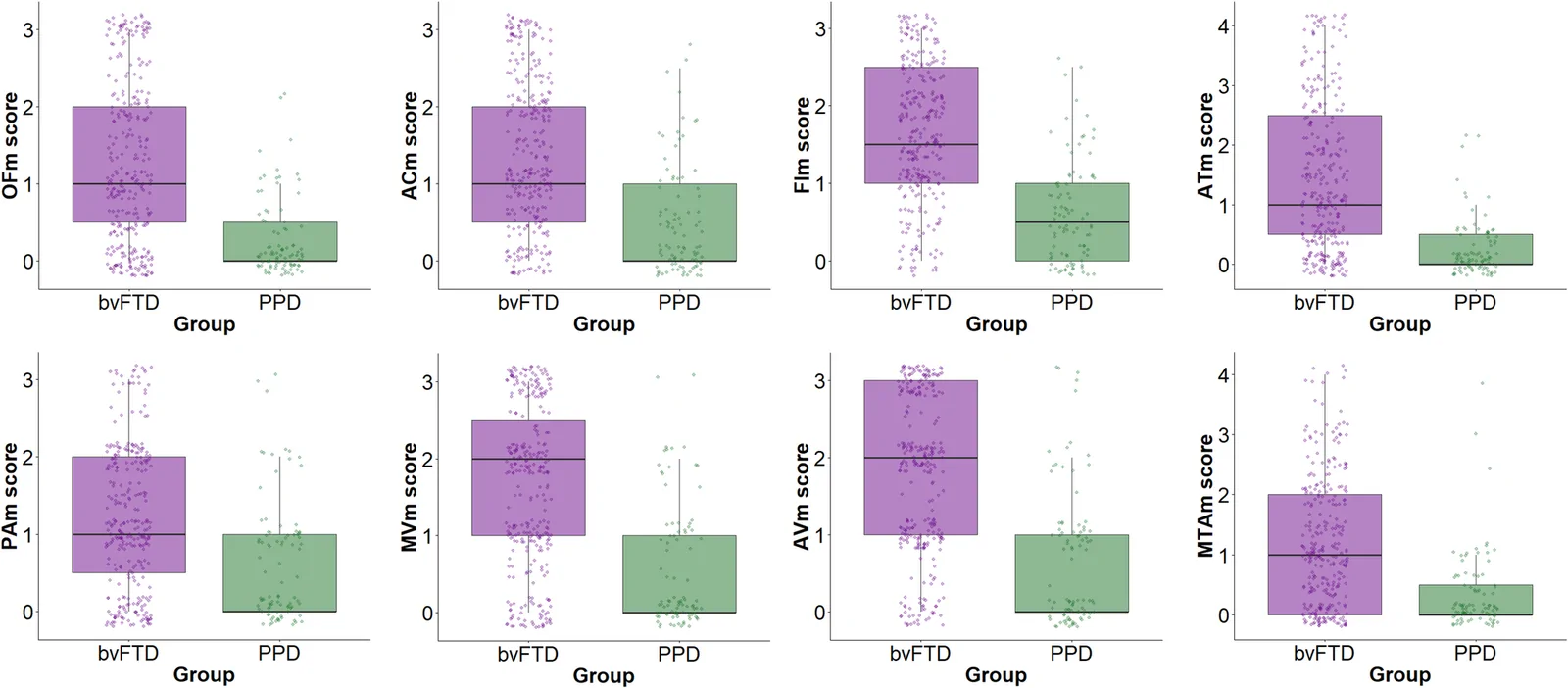
Boxplots showing the differences between the bvFTD (purple) and the PPD (green) group in each visual rating scales (OFm: mean Orbitorontal; ACm: mean Anterior Cingulate; ATm: mean Anterior Temporal; FIm: mean Fronto-Insula; MTAm: mean Medial Temporal; PAm: mean Posterior; MVm: mean Mean Ventricular; AVm: mean Axial Ventricular). The central line of the box displays the median values, the box limits represent the interquartile range (25th–75th percentiles), and whiskers extend to 1.5× the interquartile range. Individual data points are displayed as diamonds

Mean hemispheric scores across all the VRS were significantly lower in PPD compared to bvFTD (Table 2 Fig. 2), reflecting higher brain atrophy in the bvFTD group. A significant but small effect of disease duration emerged only for the MTAm (β = 0.07, SE = 0.02, *p* = 0.003) and ATm (β = 0.06, SE = 0.02, *p* = 0.02) scales, indicating that longer disease duration was associated with higher atrophy scores, and suggesting a domain-specific relationship rather than a global effect of disease progression. Group differences were not significantly influenced by sex (*p* > 0.05) and remained statistically significant after correcting for multiple comparisons (adjusted *p* < 0.008). Furthermore, results were highly consistent between the groups across the left and the right hemispheres, with comparable effect sizes (i.e., odds ratio), indicating no evidence of lateralized effects (Table S2, Supplementary Material).

**Table 2:** The mean and standard deviation in parentheses of the mean score between the left and right hemisphere in each visual rating scale

OFm	bvFTD	PPD	Group comparison	*R* ^2^
	1.26 (1.06)	0.261 (0.472)	β = -1.08, SE = 0.14, t = -7.8, *p* < 0.001	0.20
ACm	1.43 (0.993)	0.511 (0.703)	β = -0.96, SE = 0.14, t = -7.06, *p* < 0.001	0.17
ATm	1.41 (1.25)	0.245 (0.454)	β = -1.14, SE = 0.16, t = -8.79, *p* < 0.001	0.25
FIm	1.66 (0.870)	0.620 (0.656)	β = -1.1, SE = 0.11, t = -9.2, *p* < 0.001	0.26
MTAm	1.20 (1.07)	0.326 (0.644)	β = -1.05, SE = 0.14, t = -7.36, *p* < 0.001	0.19
PAm	1.19 (0.908)	0.630 (0.791)	β = -0.63, SE = 0.13, t = -4.7, *p* < 0.001	0.08
MVm	1.61 (1.04)	0.554 (0.793)	β = -1.13, SE = 0.14, t = -7.93, *p* < 0.001	0.20
AVm	1.73 (1.02)	0.701 (0.874)	β = -1.11, SE = 0.14, t = -7.71, *p* < 0.001	0.19

Mean Orbitofrontal (OFm), mean Anterior Cingulate (ACm), mean Anterior Temporal (ATm), mean Fronto-Insula (FIm), mean Medial Temporal (MTAm), mean Posterior scale (PAm), mean Mean Ventricular scale (MVm), and mean Axial Ventricular (AVm)

To assess whether non-imaging variables could alone discriminate between groups, a logistic regression model including age at onset and MMSE score was performed. Age at onset was not significantly associated with group classification (*p* = 0.37), whereas MMSE showed a significant but modest association with the diagnosis (β = 0.136, OR ≈ 1.15, *p* = 0.005), with lower MMSE scores increasing the likelihood of bvFTD group. Overall, the MMSE contribution to group discrimination was limited.

To assess whether group differences in VRS scores were independent of disease duration and MMSE, linear regression models were fitted for the mean scores of each scale. Diagnostic group remained the only significant predictor across ACm (β = -0.92, SE = 0.14, *p* < 0.001), PAm (β = -0.59, SE = 0.14, *p* < 0.001), MVm (β = -1.07, SE = 0.14, *p* < 0.001), and AVm (β = -1.1, SE = 0.14, *p* < 0.001) scales, while neither disease duration nor MMSE contributed significantly to the model (ps > 0.05). MMSE showed small but significant effects in the OFm scale (group: β = -1.02, SE = 0.14, *p* < 0.001; MMSE: β = -0.04, SE = 0.015, *p* = 0.008), and in the FIm scale (group: β = -1.00, SE = 0.11, *p* < 0.001; MMSE: β = -0.04, SE = 0.010, *p* = 0.001), where lower cognitive performance was associated with higher VRS scores. Disease duration showed a small but significant effect in the ATm scale (group: β = -1.34, SE = 0.16, *p* < 0.001; disease duration: β = 0.06, SE = 0.02, *p* < 0.001), and, together with the MMSE score, in the MTAm scale (group: β = -0.97, SE = 0.14, *p* < 0.001; MMSE: β = -0.043, SE = 0.02, *p* = 0.003; disease duration: β = 0.07, SE = 0.022, *p* = 0.001). However, across all models, diagnostic group consistently emerged as the strongest predictor.

#### VRS Diagnostic performance

ROC analyses were performed to evaluate the diagnostic accuracy of each scale (Table 3). All scales showed good discrimination between bvFTD and PPD, with AUC values ranging from 0.683 (PAm) to 0.812 (FIm). The cutpoint for calculating sensitivity and specificity (Youden Index) was 1 for all the scales included. The FIm scale achieved the highest sensitivity (84.5%), indicating superior ability to detect bvFTD cases.

**Table 3:** The diagnostic performance of the visual scales averaged for the hemisphere

OFm	Cutpoint	Sensitivity (%)	Specificity (%)	PPV (%)	NPV (%)	AUC
	1	61.95	82.8	92	40.53	0.782
ACm	1	71.72	72.04	89.12	44.37	0.767
ATm	1	61.62	89.25	94.82	42.13	0.794
FIm	1	84.51	64.52	88.38	56.6	0.812
MTAm	1	60.27	82.8	91.79	39.49	0.754
PAm	1	68.69	56.99	83.61	36.3	0.683
MVm	1	78.45	63.44	87.27	47.97	0.774
AVm	1	84.18	52.69	85.03	51.04	0.765

Positive Predictive Value (PPV), Negative Predictive Value (NPV), Area Under the Curve (AUC), mean Orbitofrontal (OFm), mean Anterior Cingulate (ACm), mean Anterior Temporal (ATm), mean Fronto-Insula (FIm), mean Medial Temporal (MTAm), mean Posterior (PAm), mean Mean Ventricular (MVm), and mean Axial Ventricular (AVm)

#### Composite score

A logistic regression model including mean VRS scores identified OFm (*p* = 0.008), ATm (*p* < 0.001), and FIm (*p* = 0.008) as significant predictors of diagnosis. The model showed good fit (χ²_(385)_ = 7.896, *p* = 0.005; McFadden’s R² = 0.31), with no significant contribution of sex or age at MRI. A composite score derived from the sum of OFm, ATm, and FIm demonstrated improved diagnostic performance (AUC = 0.861; Table 4).

**Table 4:** The ROC analysis for the composite score of the mean Orbitofrontal (OFm), mean Anterior Temporal (ATm), and mean Fronto-Insula (FIm)

Cutpoint	Sensitivity (%)	Specificity (%)	PPV (%)	NPV (%)	AUC
4	81.82%	77.17%	92.05%	56.8%	0.861
5	75.76%	82.61%	93.36%	51.35%	0.861
6	68.69%	86.96%	94.44%	46.24%	0.861

Lastly, density plots were then used to characterize the underlying distribution of anterior atrophy scores and the composite score across diagnostic groups, showing substantial overlap below the cut-offs and progressively greater separation at higher values (Figure S2, Supplementary Material).

#### The best model for bvFTD and PPD differentiation

**Fig. 3:**
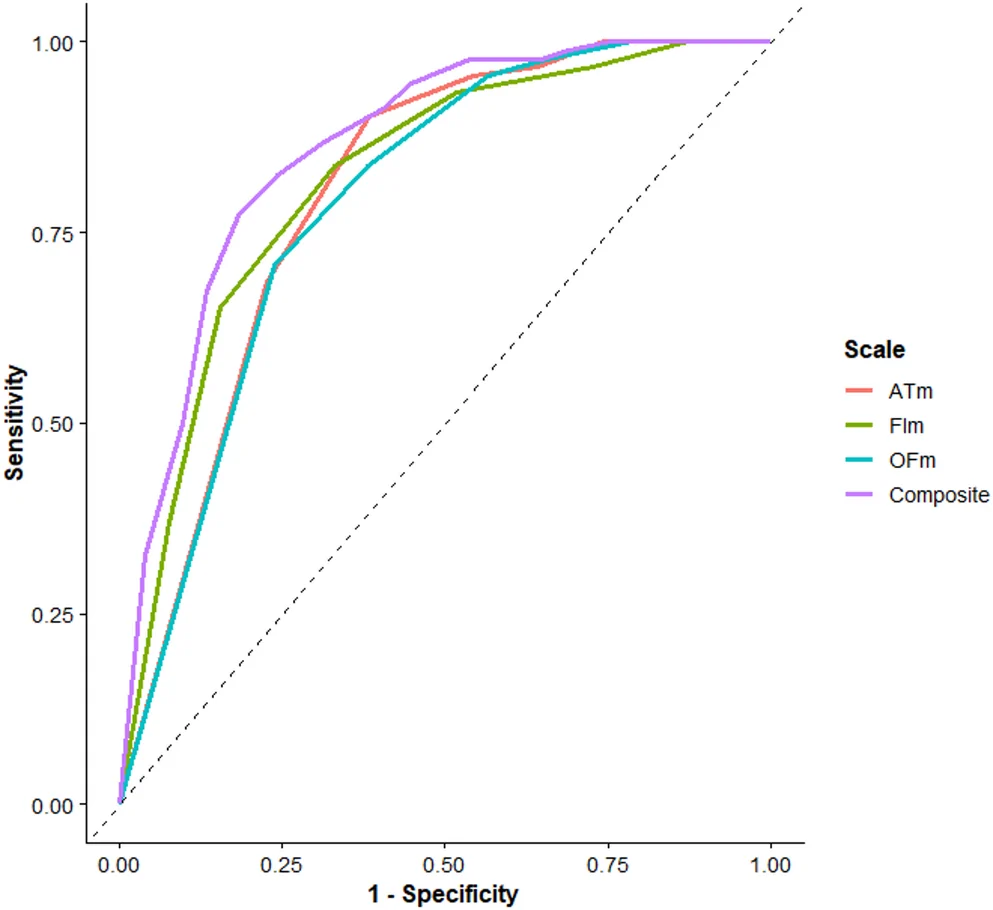
The comparison of the ROC curves combined. ATm is shown in red, FIm in green, OFm in light blue, and the composite score obtained by the sum of ATm, FIm and OFm scores in purple

The DeLong’s test (DeLong et al. 1988) revealed that the composite score achieved significantly higher diagnostic accuracy compared to OFm (*p* < 0.001), to ATm (*p* < 0.001), and to the FIm (*p* = 0.001) scale (Fig. 3). No significant differences were observed between the individual scales (ATm vs. FIm, ATm vs. OFm, FIm vs. OFm; p_s_ > 0.05), indicating comparable performance when used independently.

## Discussion

The aim of this pilot study was to assess the discriminative strength of brain MRI visual rating scales for the differential diagnosis between sporadic bvFTD and late-onset PPD patients. We found that the visual rating scales based on MRI reported a higher accuracy (81.8%) in distinguishing bvFTD from PPD compared the standard diagnostic impression (76.9%). Among the scales included, the FI scale showed the highest performance in comparison to the other scales that were tested, increasing the sensitivity compared to standard radiological inspection from 75.4% to 84.5%. The combination of FI, AT and OF, which were the visual rating scales with the highest prediction ability (86.1%), only slightly outperformed the diagnostic accuracy of the single scales. On the other hand, the posterior scale seemed less useful. Importantly, visual rating scale scores remained strongly associated with diagnostic group even after adjusting for disease duration and MMSE, which showed only limited and scale-specific effects. This may suggest that these measures capture structural brain changes that are not fully accounted for by global cognitive impairment, indicating that cognitive measures alone may be insufficient to fully characterize the two groups. This supports the potential contribution of visual rating scales to the characterization of disease-related neurodegeneration.

It has already been demonstrated that the quantitative assessment of frontotemporal brain atrophy has significant discriminative ability in distinguishing between autopsy-confirmed bvFTD and PPD (Coulborn et al. 2025). This work sought to address the need for clinically applicable tools that bridge the gap between quantitative research measures of brain atrophy and everyday clinical implementation (Coulborn et al. 2025). Our results are in line with a previous study (Cagnin et al. 2024) that reported that PET-FDG hypometabolism in the frontal and caudate regions may help distinguish bvFTD from PPD. In their study, the authors also assessed the visual rating scales of atrophy, in particular the Global Cortical Atrophy (GCA), the MTA and the Koedam for posterior cortical atrophy. The authors found significant differences between bvFTD and PPD in GCA and MTA scores, but not in the posterior regions (Cagnin et al. 2024), which is consistent with our findings, although the MTA did not emerge as the most informative measure for the differential diagnosis in our study. It is worth noting that the bvFTD group of the study (Cagnin et al. 2024) included some genetic cases (6 out of 37), which may present different atrophy patterns compared to sporadic forms. Moreover, the authors did not include visual rating scales specifically focused on frontal regions, the insula, or the temporal pole, which are often involved in bvFTD. Lastly, the GCA scale was applied on axial views with a single score across both hemispheres and without precise anatomical references, which may limit its regional sensitivity (Cagnin et al. 2024).

Similarly, Vijverberg et al. (Vijverberg et al. 2016) investigated the differences in the scores of the GCA and MTA scales between a bvFTD group from a heterogeneous non-FTD group, comprising PPD as well as other neurological conditions. The MTA scales showed a significantly higher degree of atrophy in the FTD patients, which is consistent with our findings. Furthermore, the authors reported a sensitivity of 70% and a specificity of 93% for frontotemporal atrophy based on the MRI analyses. Our findings obtained by the implementation of the visual rating scales align with this, showing a globally moderate sensitivity and specificity of MRI-assessed frontotemporal changes for identifying bvFTD. Furthermore, the rater’s diagnostic impression also reaches moderate levels of sensitivity and specificity. These results highlight the value of structural MRI as a first-line tool in a multimodal evaluation.

Combining multiple visual rating scales can balance individual scale limitations, and enhance overall discriminative power. Thus, a previous study has proposed a composite atrophy score derived from multiple visual rating scales as practical tools to quantify frontotemporal degeneration as bedside tool (Illán-Gala et al. 2021). In particular, this study calculated bvFTD atrophy score combined ratings from the five regions frequently affected in bvFTD: the anterior cingulate, orbitofrontal, frontal insula, anterior temporal, and medial temporal lobe. The composite score obtained by the authors has shown strong diagnostic performance in distinguishing bvFTD from healthy controls, and in stratifying cases based on diagnostic confidence (high confidence vs. low confidence) (Illán-Gala et al. 2021). In this study, we adopted a data-driven approach to identify the most discriminative visual rating scales for differentiating sporadic bvFTD from PPD. Among the scales evaluated, only three, namely the OF, the AT and the FI, demonstrated the highest discriminative accuracy. These findings suggest that a simplified composite score incorporating only these three regions may provide a time-efficient yet accurate tool. Notably, similar accuracy was achieved using a single scale (i.e., FI), suggesting that FI alone may provide a rapid and practical approach to support differential diagnosis when speed and ease of use are necessary. However, in contexts where a more stringent approach is required, such as in clinical research, where precision is paramount and minimizing false positives becomes essential, the composite score may represent a more robust solution. Therefore, while the FI scale may suffice for routine clinical assessment, the full battery remains valuable in research settings.

Two newly developed visual rating scales for the ventricular enlargement were included in order to further explore the subcortical atrophy and/or global involvement. Their reproducibility and generalizability remain to be established, although both scales demonstrated a good intra-rater reliability. This result should therefore be considered preliminary, and future studies involving multiple raters with different expertise will be necessary to formally validate these scales in larger independent cohorts.

Moreover, despite significant group differences and acceptable diagnostic accuracy for the anterior visual rating scales, considerable overlap between bvFTD and PPD was observed at lower VRS scores, whereas greater separation emerged with increasing atrophy severity. This finding suggests that VRS could be more informative in cases with moderate-to-severe structural abnormalities, whereas individuals with absent or mild atrophy remain more difficult to classify. Therefore, VRS may provide valuable support for pattern recognition and diagnostic decision-making, but should be interpreted within a multimodal assessment integrating clinical and neuropsychological information.

A smaller sample of the same retrospective DIPPA cohort was recently studied using FDG-PET, and this method demonstrated 88% accuracy in differentiating bvFTD from PPD. This level of accuracy is higher than that found in the current study, supporting the clinical practice of using FDG-PET as a second neuroimaging step for ambiguous cases in which there are no clear frontotemporal atrophy findings on MRI (Rue et al. 2025).

A key strength of the study was the relatively large sample size, drawn from five different centres, together with the applicability of these findings to real-world radiological and clinical settings, where clinical information is often limited or incomplete. This reflects a pragmatic, naturalistic scenario in which neuroradiologists may need to rely primarily on imaging features when clinical data are insufficient to clearly differentiate between groups. However, this study has several limitations. First, visual ratings were conducted by a single expert rater, and the diagnostic performance observed in the present study may have been influenced, at least in part, by the expertise of the rater in both bvFTD and the application of visual rating scales. Therefore, the generalizability and replicability of these findings to routine clinical practice should be confirmed in studies involving multiple independent raters with different backgrounds and levels of disease-specific expertise. However, intra-rater reliability was high, and the rater’s performance has been shown to be consistent with other trained raters in previous studies (Fumagalli et al. 2018, Falgàs et al. 2024, Harper et al. 2016, Fumagalli et al. 2020). Second, grouping patients with various primary psychiatric disorders together may also be seen as a limitation, but it mirrors real-life diagnostic uncertainty. Notably, the cohort was predominantly composed of patients with major depressive disorder, with other psychiatric diagnoses being less frequent. Moreover, the study did not include a healthy control group, which would have been valuable to better contextualize the degree of atrophy observed in the PPD group, and the groups were unequal in sample size. Consequently, predictive values (i.e., PPV and NPV) should be interpreted within the context of the study sample, as these metrics are influenced by disease prevalence and may vary across clinical settings. By contrast, sensitivity, specificity and AUC are more stable and may provide a more robust estimate of diagnostic performance. In addition, although the sample was restricted to well-characterized bvFTD cases, substantial inter-individual variability in regional atrophy patterns and hemispheric asymmetry may still be present within this diagnostic category. This intrinsic heterogeneity may have contributed to the overlap observed between diagnostic groups in visual rating scale scores. Third, while the study design aimed to reflect real-world clinical practice, clinicians typically have access to additional patient information, which may both enhance diagnostic accuracy and introduce bias for the rating of brain atrophy. Forth, the retrospective multicenter design based on routine clinical data may constrain generalizability, particularly due to the incomplete and non-systematic collection of clinically relevant information, such as the characterization of psychiatric symptoms or more domain-specific cognitive assessments (de Boer et al. 2025), which were not consistently available across cohorts. In addition, heterogeneity in MRI acquisition parameters may have introduce unquantified measure of noise (Fischbach-Boulanger et al. 2018), particularly in the assignment of borderline scores (e.g., distinguishing between absent and mild atrophy). Although visual rating scales are generally considered less dependent to scanner-related variability than automated volumetric approaches (Harper et al. 2016), this factor should be taken into account when interpreting the findings. However, a prospective longitudinal cohort from the DIPPA study is currently underway to address these in future analyses (de Boer et al. 2024). Lastly, a degree circularity should be acknowledged, as the criteria for probable bvFTD require atrophy and/or hypometabolism in the frontal and/or temporal lobes. Consequently, the visual rating scales evaluated in this study are not entirely independent of the clinical diagnosis. However, patient diagnoses were established through a multidisciplinary assessment integrating clinical history, neuropsychological evaluation, and imaging information and were not based on the VRS themselves. Therefore, the observed group differences and diagnostic accuracy estimates should be interpreted taking this potential dependency into account.

To conclude, visual rating scales for brain atrophy may discriminate between sporadic bvFTD and PPD patients, although their accuracy shows specificity to certain brain regions, supporting the need of a multimodal approach for the differential diagnosis. A composite score of three visual rating scales (fronto-insula, orbitofrontal and anterior temporal) could improve diagnostic accuracy compared to using individual scales alone. Furthermore, while the standard radiological inspection accurately discriminated between the two groups, the fronto-insula rating scale seemed more effective.

## Supplementary Information

Below is the link to the electronic supplementary material.

Supplementary Material 1

## Data Availability

The datasets used and/or analysed during the current study are available from the corresponding author on reasonable request.
