# Supplementary material for: Visual rating scales of atrophy differentiate sporadic behavioral variant frontotemporal dementia from primary psychiatric disorder: DIPPA study
Source: J Neural Transm (Vienna). 2026 Jul 2;133(9):2623–34. doi: 10.1007/s00702-026-03223-y (PMC13687229; doi:10.1007/s00702-026-03223-y)
Supplement: Supplementary file 1 [file 702_2026_3223_MOESM1_ESM.docx]

**Supplementary Material**

| **Site** | **Magnetic field** | **Scanner make & model** | **Coil** | **slices** | **TR** | **TE** | **FOV** | **Voxel size (mm)** | **Flip angle** |
| --- | --- | --- | --- | --- | --- | --- | --- | --- | --- |
| Italy | 3T | Philips Achieva dStream | 32 channel head | 185 | 9.8 | 4.6 | 256x256 | 1x1x1 | α = 8 |
| Canada | 3T | Siemens Magnetom Prisma |  | 192 | 2300 | 2.98 | 256x256 | 1x1x1 | α = 8 |
| Germany | 3T | Philips Ingenia | 32 channel head | 208 | 6.8 | 3.2 | 256x256 | 1.1x1.1x  1.1 | α = 8 |
| Australia | 3T | GE Discovery MR 750 | 8 channel head | 200 | 6.6 | 2.4 | 256x256 |  |  |
| Australia | 3T | Philips Achieva Gyroscan | 8 channel head | 200 | 5.8 | 2.6 | 256x256 | 1x1x1 | α = 8 |
| Netherlands | 1T | Siemens Magnetom Impact |  |  | 15 | 7 |  | 1x1x1.5 | α = 15 |
| Netherlands | 1.5T | Siemens Vision |  |  | 15 | 7 |  | 0.98x0.98x1.5 | α = 8 |
| Netherlands | 1.5T | Siemens Sonata |  |  | 2700 | 3.97 |  | 1x1x1.5 | α = 8 |
| Netherlands | 1.5T | Siemens Avanto |  |  | 2700 | 5.2 |  | 1x1x1.5 | α = 8 |
| Netherlands | 1.5T | GE SignaHDxt |  |  | 12.4 | 5.17 |  | 0.98x0.98x1.5 | α = 12 |
| Netherlands | 3T | GE SignaHDxt |  |  | 8 | 3 |  | 0.98x0.98x1 | α = 12 |
| Netherlands | 3T | Toshiba Titan |  |  | 9.5 | 3.2 |  | 1x1x1 | α = 12 |
| Netherlands | 3T | Philips Ingenuity |  |  | 8 | 4 |  | 1x1x1 | α = 12 |

**Table S1**. MRI acquisition parameters for each centre.

**Figure S1**. Reference images of the visual rating scales used in the protocol. OF: orbitofrontal; AC: anterior cingulate; AT: anterior temporal; FI: Fronto-insula; MTA: Medial temporal; PA: Posterior; MV: mean ventricular; AV: Axial ventricular.

|  | **bvFTD** | **PPD** | **Group comparison** | **OR** |
| --- | --- | --- | --- | --- |
| **OFR** | 1.13 (1.1) | 0.25 (0.53) | β = -2.15, SE = 0.33, z = -6.48, p < 0.001 | 0.12 |
| **OFL** | 1.30 (1.1) | 0.27 (0.49) | β = -2.22, SE = 0.32, z = -6.95, p < 0.001 | 0.11 |
| **ACR** | 1.39 (1.06) | 0.54 (0.83) | β = -1.56, SE = 0.29, z = -5.47, p < 0.001 | 0.21 |
| **ACL** | 1.47 (1.1) | 0.48 (0.73) | β = -2.04, SE = 0.3, z = -6.86, p < 0.001 | 0.13 |
| **ATR** | 1.43 (1.37) | 0.24 (0.52) | β = -2.72, SE = 0.36, z = -7.58, p < 0.001 | 0.07 |
| **ATL** | 1.39 (1.3) | 0.25 (0.51) | β = -2.29, SE = 0.32, z = -7.13, p < 0.001 | 0.10 |
| **FIR** | 1.66 (1.0) | 0.54 (0.7) | β = -2.56, SE = 0.32, z = -7.86, p < 0.001 | 0.08 |
| **FIL** | 1.66 (0.96) | 0.7 (0.77) | β = -1.89, SE = 0.29, z = -6.48, p < 0.001 | 0.15 |
| **MTAR** | 1.25 (1.21) | 0.37 (0.71) | β = -1.96, SE = 0.31, z = -6.38, p < 0.001 | 0.14 |
| **MTAL** | 1.15 (1.1) | 0.28 (0.65) | β = -2.28, SE = 0.33, z = -6.97, p < 0.001 | 0.10 |
| **PAR** | 1.2 (0.95) | 0.64 (0.79) | β = -1.29, SE = 0.28, z = -4.59, p < 0.001 | 0.28 |
| **PAL** | 1.19 (0.93) | 0.62 (0.81) | β = -1.28, SE = 0.28, z = -4.53, p < 0.001 | 0.28 |
| **MVR** | 1.58 (1.1) | 0.54 (0.8) | β = -1.91, SE = 0.29, z = -6.55, p < 0.001 | 0.15 |
| **MVL** | 1.65 (1.04) | 0.57 (0.8) | β = -2.15, SE = 0.3, z = -7.2, p < 0.001 | 0.12 |
| **AVR** | 1.71 (1.1) | 0.81 (0.88) | β = -1.88, SE = 0.29, z = -6.51, p < 0.001 | 0.15 |
| **AVL** | 1.76 (1.02) | 0.7 (0.87) | β = -2.13, SE = 0.3, z = -7.1, p < 0.001 | 0.12 |
| **Table S2**. The mean and standard deviation in parentheses of the mean score in the left and right hemisphere for each visual rating scale. Regression coefficients are reported as estimates (β) with standard errors, statistical significance was assessed using Wald z-tests, and the odds ratio (OR) was calculated. Abbreviations: Orbitofrontal (OF), Anterior Cingulate (AC), Anterior Temporal (AT), Fronto-Insula (FI), Medial Temporal (MTA), Posterior scale (PA), Mean Ventricular scale (MV), Axial Ventricular (AV), left (L), and right (R), odds ratio (OR). | | | | |

**Figure S2.** Density distribution. (**A**) Anterior-temporal mean (ATm); (**B**) Orbito-frontal mean (OFm); (**C**) fronto-insular mean (FIm); (**D**) composite score in bvFTD (green) and PPD (purple) patients. The curves illustrate the distribution of the VRS scores within each diagnostic group and the degree of overlap between groups.
